# Correction: Going the distance: Neocentromeres make long-range contacts with heterochromatin

**DOI:** 10.1083/jcb.20181117201102019c

**Published:** 2019-02-04

**Authors:** Shannon M. McNulty, Beth A. Sullivan

Vol. 218, No. 1, January 7, 2019. 10.1083/jcb.201811172.

Following publication, the *JCB* staff noted errors in the in-text citations and order of papers in the reference list that were introduced during production of the article. The authors were alerted of these inaccuracies and we apologize to our readers for these errors.

Both the HTML and PDF versions of the article have been corrected. These errors appear only in print and PDF versions downloaded on or before January 10, 2019.

